# Urine albumin and serum uric acid are important determinants of serum 25 hydroxyvitamin D level in pre-dialysis chronic kidney disease patients

**DOI:** 10.1080/0886022X.2018.1563552

**Published:** 2019-06-25

**Authors:** Ahmed Fayed, Mahmoud M. El Nokeety, Ahmed A. Heikal, Khaled M. Sadek, Hany Hammad, Dina O. Abdulazim, Mona M. Salem, Usama A. Sharaf El Din, on behalf of the Vascular Calcification Group (VCG)

**Affiliations:** a Nephrology Unit, Internal Medicine Department, School of Medicine, Cairo University, Manial, Egypt;; b Internal Medicine Department, School of Medicine, Cairo University, Manial, Egypt;; c Rheumatology and Rehabilitation Department, School of Medicine, Cairo University, Manial, Egypt;; d Endocrinology Unit, Internal Medicine Department, School of Medicine, Cairo University, Manial, Egypt

**Keywords:** CKD, vitamin D deficiency, 25 hydroxy vitamin D, serum phosphorus, urine albumin excretion, uric acid, parathormone

## Abstract

Low serum 25 hydroxyvitamin D (25 OH D) is common among chronic kidney disease (CKD) patients. This cross-sectional study is looking for the different factors associated with serum 25 OH D among pre-dialysis CKD. 1624 adult stage 3–5 CKD patients were studied beside 200 normal control subjects. All candidates were tested for body mass index (BMI), estimated glomerular filtration rate (eGFR), calcium (Ca), phosphorus (P), parathormone (PTH), 25 OH D, albumin, and uric acid (UA), and urine albumin/creatinine ratio (ACR). Multivariate linear regression analysis was done to determine predictors of 25 OH D. 98.6% of CKD patients have inadequate level of 25 OH D vs 48% of normal subjects. Serum 25 OH D was significantly lower in CKD patients (mean ± S.D = 16.54 ± 5.8 vs 37.79 ± 3.58 ng/mL for CKD vs control group respectively, *p* < .001). Serum level of 25 OH D has significant positive correlation with Ca (*r* = 0.337, *p* < .001), and significant negative correlation with P, PTH, UA, and ACR (*r* = −0.440, −0. 679, −0.724, and −0.781respectively, *p* < .001 in all). The independent predictors of 25 OH D were Ca, P, UA, PTH, and ACR (R square = 0.7, β = −0.087, −0.226, −0.313, −0.253, and −0.33 respectively, *p* < .001 in all). In conclusion, pre-dialysis CKD patients frequently suffer low 25 OH D. Among the different abnormalities related to CKD, urine albumin excretion rate and UA are the most important predictors of 25 OH D in these patients.

## Introduction

25 OH D is the most appropriate diagnostic test for Vitamin D status. Serum level above 30 ng/mL is considered sufficient, while a level between 20 and 30 ng/mL is considered as vitamin D insufficiency and level below 20 ng/mL denotes vitamin D deficiency [1]. Vitamin D deficiency may underlie various diseases including cardiovascular, neoplastic, infectious, metabolic, and autoimmune diseases. It may also accelerate progression of CKD [2,3]. Most of the studies that looked for vitamin D status among CKD patients have disclosed significant increase in prevalence and severity of vitamin D deficiency among these patients compared to normal subjects. Besides the well-known environmental, demographic, and nutritional causes, different other factors were incriminated in association with vitamin D deficiency. These factors include dietary restriction, diabetes and increased body mass index [4]. UAE was also appointed in some studies as another factor associated with decreased 25 OH D [5,6]. In spite of the high prevalence of low 25 OH among CKD patients, there is marked discrepancy in the prevalence in between different studies. While 77% of CKD patients in the State of Louisiana, one of the sunny areas in USA, had suboptimal levels of 25 OH D [7], only 39.6% of similar patients in the sunnier Italy were considered vitamin D-insufficient [8]. In addition, there is marked variability in the associated factors in these studies. While some studies reported significant association with age [9–11] other studies failed to show similar association. Similar discrepancies are observed regarding association with body weight [11,12], and diabetic status [7,11,13–15]. Moreover, most of these studies failed to find an association with GFR. In the present study, we recruited patients living in different areas of Egypt and looked for all possible demographic and comorbid factors that could have an association with serum 25 OH D level.

## Patients and methods

1624 CKD patients (688 male and 936 female) were included. The age of this group ranged between 16 and 55 years. The underlying causes of their CKD are summarized in Table 1. 271 cases (16.7%) were in stage 3, 1290 (79.4%) in stage 4 and 63 (3.9%) in stage 5. For comparative analysis, 200 normal control subjects were included.

**Table 1.** Etiology of chronic kidney disease in the patient group.

Etiology	No. (%)	Etiology	No. (%)
Diabetes mellitus	527 (32.5%)	Systemic hypertension	379 (23.3)
Ch. Interstitial nephritis	287 (17.6%)	Ch.glomerulonephritis	56 (3.4%)
Obstructive uropathy	158 (9.8%)	Polycystic Kidney	82 (5%)
Reflux nephropathy	39 (2.4%)	Hereditary nephropathy	14 (0.86%)

After getting the written consent, every candidate was clinically examined and a blood and urine samples were collected. Serum 25 OH D was assessed using HPLC [16]. Intact PTH serum level was determined by enzyme-amplified sensitive immunoassay (Roche Diagnostics, IN, USA). eGFR was calculated using MDRD equation [17].

Statistical analysis was done by SPSS computer package. Data were first subjected for tests of normality. Most of the variables were not normally distributed as derived from normality test results. Data were presented as mean ± standard deviation as well as median and interquartile range (IQR). Mann–Whitney test was used instead of independent *t*-test to compare groups. Spearman univariate analysis was used to find out the possible significant associations between serum 25 OH D and the different studied parameters. A multiple linear regression model was used to analyze predictors of 25 OH D. The predictors that are included in the model are the variants that showed statistical significance in univariate analysis, namely, serum Ca, P, PTH, UA, albumin, and urine ACR. The adjusted R-squared of the final model was 0.7.

We then subdivided the patient group according to urine albumin excretion and serum uric acid in order to compare the different demographic and laboratory variables within these subgroups. Mann–Whitney test was used to determine whether there are any statistically significant differences within these tertiles.

## Results

**Figure 1.**
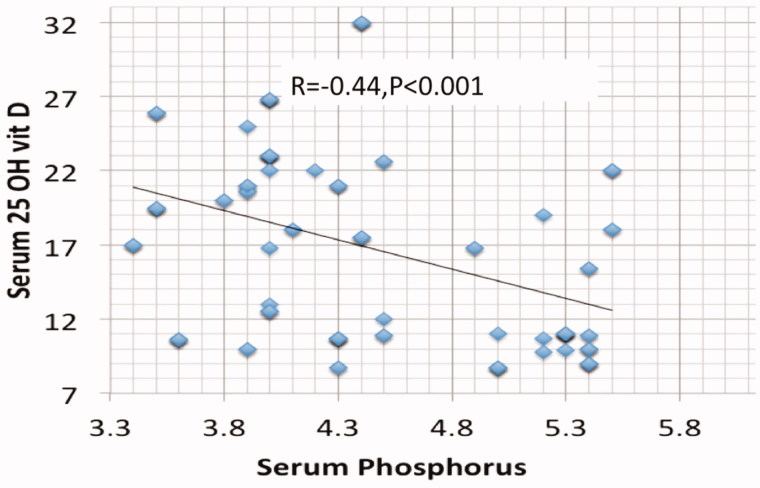
Spearman Correlation between serum phosphorus and serum 25 hydroxyvitamin D.

**Figure 2.**
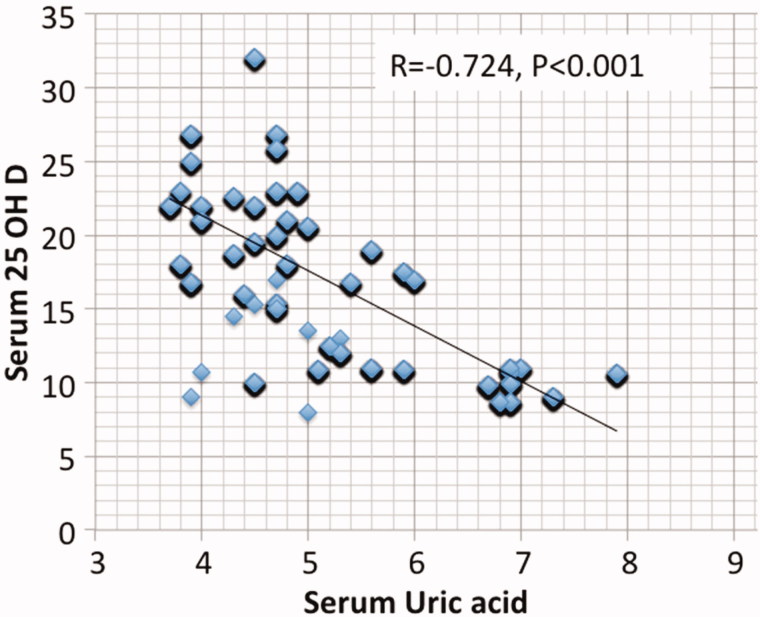
Spearman Correlation between serum uric acid and serum 25 hydroxyvitamin D.

**Figure 3.**
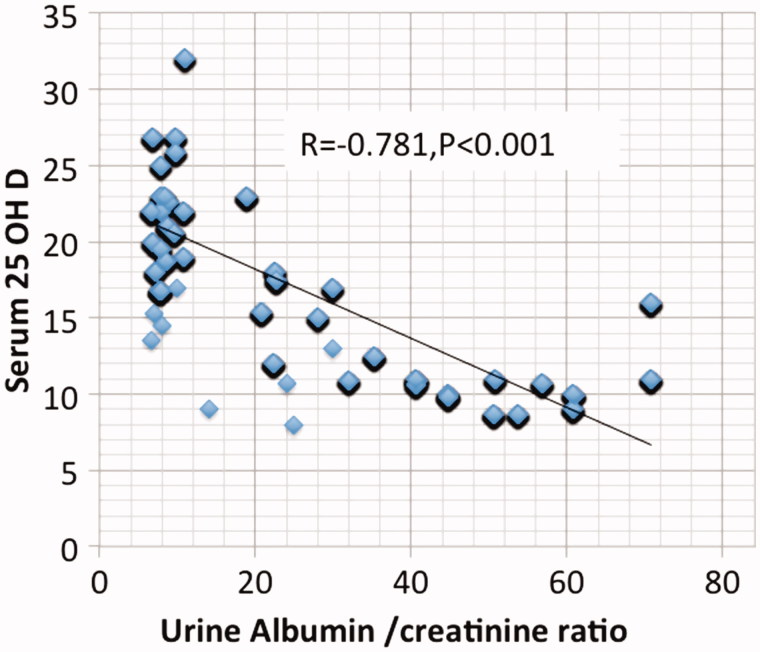
Spearman Correlation between urine albumin and serum 25 hydroxyvitamin D.

**Figure 4.**
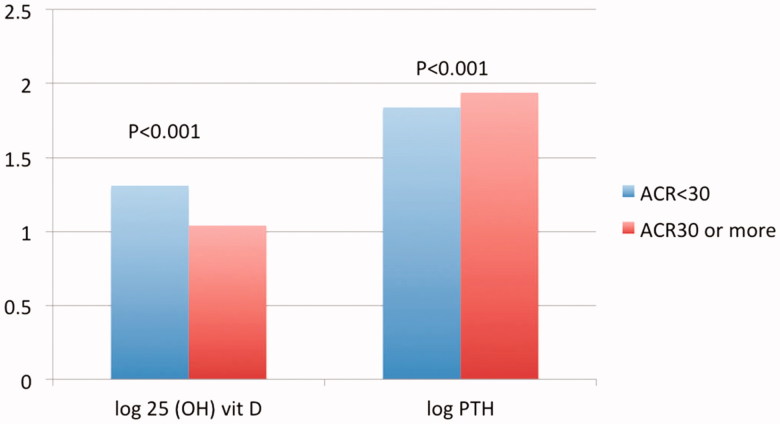
Serum 25 (OH) vitamin D and parathormone according to urine albumin excretion.

**Figure 5.**
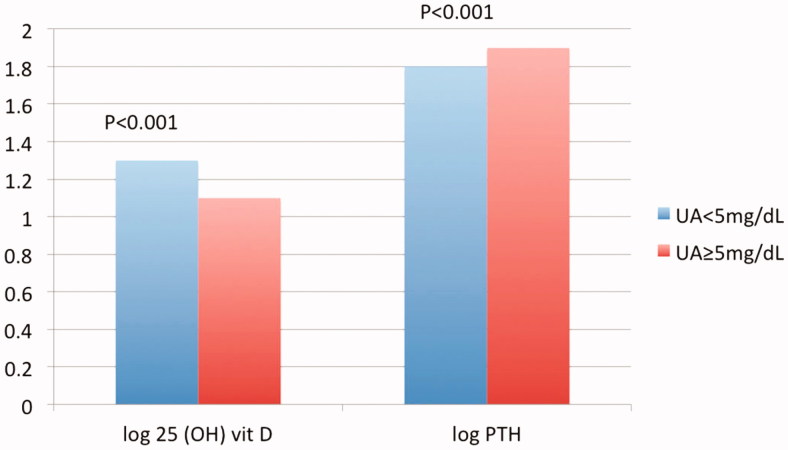
Serum 25 (OH) vitamin D and parathormone according to serum uric acid.

Results are summarized in Tables 1–6 and Figures 1–5. 98.6% of CKD patients (1602 out of 1624) had suboptimal 25 OH D levels (<30 ng/mL) in comparison to 48% of the normal control subjects (Table 2). There is no significant association between 25 OH D and either age, BMI, or estimated GFR. On the other hand, a highly significant negative correlation (Two-tailed probability is significant at 0.01 level) is observed between 25 OH D and serum P (*r* = −0.44), serum PTH (*r* = −0.679), serum UA (*r* = −0.724) and urine ACR (*r* = −0.781). Serum Ca (*p* < .001), P (*p* < .001), PTH (*p* < .001), UA (*p* < .001), and ACR (*p* < .001) were independently related to 25 OH D in a multivariate linear regression analysis. ACR was found to be the most significant predictor (beta -0.33) followed by UA (beta- 0.313).

**Table 2.** Demographic and laboratory characteristics in patient versus control groups.

	CKD group *N* = 1624		Control group *N* = 200		
	Mean ± SD	Median(IQR)	Mean ± SD	Median(1QR)	*p* value
Age (years)	30.62 ± 8.74	28 (24–37)	35.5 ± 4.79	37 (31–40)	<.001
BMI (kg/m^2^)	23.19 ± 2.71	23.5 (21.8–25)	22.78 ± 2.62	22.6 (22–25)	.004
BUN (mg%)	18.51 ± 3.92	19 (15–22)	13.23 ± 2.43	13 (11–16)	<.001
Creatinine (mg%)	3.01 ± 0.57	2.9 (2.6–3.4)	0.76 ± 0.15	0.7 (0.7–0.8)	<.001
Albumin (gm%)	3.52 ± 0.13	3.5 (3.4–3.6)	4.09 ± 0.21	4.1 (3.9–4.2)	<.001
eGFR(ml/min/1.73m^2^)	23.43 ± 6.66	22.7 (18.1–26.9)	111.13 ± 28.96	113.3 (85.4–138.6)	<.001
Calcium (mg%)	8.09 ± 0.28	8 (7.8–8.3)	9 ± 0.29	8.9 (8.8–9.3)	<.001
Phosphorus (mg%)	4.5 ± 0.64	4.3 (4–5.2)	3.68 ± 0.25	3.6 (3.5–4)	<.001
PTH (pg/ml)	76.37 ± 14.04	77.8 (69–89.4)	47.79 ± 4.49	47 (45.9–50.5)	<.001
25OH Vit D (ng/ml)	16.54 ± 5.8	16.8 (10.9–21)	37.79 ± 3.58	38.7 (35.9–40.7)	<.001
ACR (mg/gm)	27.32 ± 20.5	22.4 (8.5–44.7)	8.43 ± 2.57	7.9 (7.8–8.9)	<.001
Uric acid (mg%)	5.28 ± 1.13	4.9 (4.5–6)	4.67 ± 0.52	4.5 (4.1–5.2)	<.001

Mann–Whitney test is used; CKD: chronic kidney disease; BMI: body mass index; BUN: blood urea nitrogen; eGFR: estimated glomerular filtration rate; PTH: parathyroid hormone; ACR: albumin/ creatinine ratio.

**Table 3.** Correlation of 25 (OH) vitamin D with different variables in CKD group.

	25OH Vit D(ng/mL)	
	*r*	*p* value
Age	−0.023	.347
BMI	−0.002	.939
Serum Albumin	0.071^a^	.004
eGFR (ml/min/1.73m^2^)	0.003	.911
Serum Calcium	0.337^a^	<.001
Serum Phosphorus	−0.440^a^	<.001
PTH (pg/ml)	−0.679^a^	<.001
ACR	−0.781^a^	<.001
Uric acid	−0.724^a^	<.001

a Correlation is significant at the 0.01 level (2-tailed). Spearman non-parametric test is used. PTH: parathyroid hormone; ACR: albumin/ creatinine ratio.

**Table 4.** Multiple linear regression for predictors of vitamin D level.

(Constant)	Beta	R Square	*p* value
Serum Albumin	0.021	0.7	.092
Serum Calcium	−0.087		<.001
Serum Phosphorus	−0.226		<.001
PTH (pg/ml)	−0.253		<.001
ACR	−0.33		<.001
Uric acid	−0.313		<.001

Variables entered in the model were significant in the univariate analysis. PTH: parathyroid hormone; ACR: albumin/creatinine ratio.

**Table 5.** Demographic and laboratory characteristics in patient group according to urine albumin.

	Urine Albumin:creatinine ratio				
	<30 mg/g		≥30 mg/g		
	Mean ± SD	Median (IQR)	Mean ± SD	Median (IQR)	*p* value
Age (years)	30.6 ± 8.88	28 (24:37)	30.65 ± 8.56	28 (24:38)	.669
BMI (kg/m^2^)	23.21 ± 2.72	23.5 (21.8:25)	23.15 ± 2.71	23.5 (21.8:25)	.806
BUN (mg%)	18.44 ± 3.98	19 (15:22)	18.61 ± 3.82	19 (15:22)	.278
Creatinine (mg%)	3.01 ± 0.56	2.9 (2.6:3.4)	3.01 ± 0.57	2.9 (2.5:3.4)	.728
Albumin (gm%)	3.53 ± 0.13	3.5 (3.4:3.6)	3.51 ± 0.13	3.5 (3.4:3.6)	.003
eGFR(ml/min/1.73m^2^)	23.39 ± 6.58	22.8 (18.2:26.5)	23.5 ± 6.78	22.6 (18:27.1)	.967
Calcium (mg%)	8.14 ± 0.23	8.1 (8:8.3)	8.02 ± 0.33	7.8 (7.8:8.3)	<.001
Phosphorus (mg%)	4.34 ± 0.58	4.1 (4:4.5)	4.74 ± 0.65	5 (4.3:5.3)	<.001
PTH(pg/ml)	68.73 ± 12.02	70 (60:76)	87.37 ± 8.26	89.4 (82:94)	<.001
25OH Vit D(ng/ml)	20.42 ± 4.15	20.6 (17.5:23)	10.96 ± 2.08	10.7 (9.8:11)	<.001
Uric acid (mg%)	4.58 ± 0.58	4.7 (4:4.8)	6.29 ± 0.95	6.8 (5.6:6.9)	<.001

Mann–Whitney test is used; BMI: body mass index; BUN: blood urea nitrogen; eGFR: estimated glomerular filtration rate; PTH: parathyroid hormone.

**Table 6.** Demographic and laboratory characteristics in patient group according to serum uric acid.

	Serum uric acid				
	<5 mg/dL		≥5 mg/dL		
	Mean ± SD	Median (IQR)	Mean ± SD	Median (IQR)	*p* value
Age (years)	30.42 ± 8.78	28 (23:37)	30.85 ± 8.71	28 (24:38)	.233
BMI (kg/m^2^)	23.14 ± 2.73	23.5 (21.8:25)	23.24 ± 2.69	23.5 (21.8:25)	.408
BUN (mg%)	18.34 ± 4	18 (15:22)	18.7 ± 3.81	19 (16:22)	.026
Creatinine (mg%)	3.01 ± 0.57	2.9 (2.6:3.4)	3.01 ± 0.57	2.9 (2.5:3.4)	.635
Albumin (gm%)	3.52 ± 0.13	3.5 (3.4:3.6)	3.51 ± 0.13	3.5 (3.4:3.6)	.095
eGFR(ml/min/1.73 m^2^)	23.46 ± 6.63	22.8 (18.2:26.6)	23.4 ± 6.71	22.5 (17.9:26.9)	.723
Calcium (mg%)	8.12 ± 0.24	8 (8:8.3)	8.06 ± 0.32	7.9 (7.8:8.3)	<.001
Phosphorus (mg%)	4.34 ± 0.63	4 (4:4.5)	4.68 ± 0.6	4.9 (4.3:5.3)	<.001
PTH (pg/ml)	67.86 ± 12.15	69 (59:76)	86.13 ± 8.73	88 (78:94)	<.001
25OH Vit D (ng/ml)	20.67 ± 4.35	21 (18:23)	11.81 ± 2.94	10.9 (9.9:12.5)	<.001
ACR (mg/gm)	15.13 ± 15.49	8.7 (7.9:14)	41.27 ± 16.17	40.8 (30:53.7)	<.001

Mann–Whitney test is used; BMI: body mass index; BUN: blood urea nitrogen; eGFR: estimated glomerular filtration rate; PTH: parathyroid hormone; ACR: albumin/creatinine ratio.

Table 5 and Figure 4 highlight values of serum 25 OH D and PTH within the created subgroups according to ACR while Table 6 and Figure 5 highlight the same parameters according to the level of serum UA.

## Discussion

The very high prevalence of low 25 OH D levels in the present trial casts doubt about the role of nutrition and environment as determinants of 25 OH D among CKD patients. This study has recruited CKD patients that were not yet kept on a restricted diet. In addition, the current study was run in Egypt, a more sunny country compared to Italy and Louisiana State. Studies performed in these two regions have shown much lower prevalence of low 25 OH D [7,8]. Meanwhile, the lack of gender difference observed in the current study reinforces this opinion. Females are less exposed to the sun in the region of current study for cultural and religious reasons. In addition, the current study failed to find out a significant impact of age or BMI on 25 OH D.

In spite of the frequent reports on the association of the diabetic state with low 25 OH D, we failed to find out similar association. A recent study of 64144 subjects with or without CKD among diabetic patients has also failed to find an impact of the diabetic stare on 25 OH D [15].

The possible relationship between serum UA and vitamin D metabolism was initially raised more than 25 years ago. BY that time, uric acid was accused to decrease serum 1,25 (OH)_2_ vitamin D level [18,19]. On the other hand, the association between high serum UA and low 25 OH D was first disclosed four years ago among elderly healthy women [20]. On the contrary, a recent study of diabetic CKD patients reported a significant association of low level of vitamin D with low serum UA [21]. In the current study, Serum UA is the 2nd most important predictor of 25 OH D. The mechanism underlying this association is not yet known. Serum UA is correlated with serum fibroblast growth factor-23 (FGF23) in kidney transplantation patients [22]. In multivariable-adjusted analyses in 1261 participants of the Health Professionals Follow-up Study, higher serum PTH, P, and UA, all were associated independently with higher FGF23 in models adjusted for age, creatinine, and other factors [23]. In 98 children with normal kidney function, multivariable analyses disclosed 25 OH D, uric acid, and phosphorus as independent predictors of C-terminal FGF23 [24]. In a cross-sectional study of 537 CKD patients, FGF23 was independently associated with UA metabolism. In addition, the fractional excretion of phosphorus showed inverse association with UA clearance. These findings suggested the possible mutual relationships between urinary P excretion and UA excretion. The authors suggested a possible role of FGF23 in regulating both renal P and UA handling in CKD patients [25]. On the other hand, a more recent animal study in rats has demonstrated that elevated PTH downregulates the intestinal and renal UA transporter ABCG2 [26]. Down-regulation of this UA transporter would enhance UA retention in CKD patients with secondary hyperparathyroidism. Based on these findings, it seems that High serum PTH and FGF23 triggered by decreased handling of P by the diseased kidneys can cause UA retention. Decreased serum 25 vit D level, commonly encountered in CKD patients can additionally stimulate PTH secretion and consequent UA retention. However, UA may have an inhibitory effect on 25 hydroxylation of vitamin D. UA is significantly associated with nonalcoholic fatty liver disease (NAFLD) in patients with or without CKD [27,28]. In addition, low 25 OH D is frequently reported in patients suffering NAFLD [29]. The liver is almost the sole source of 25 hydroxylation of vitamin D [30]. UA is also able to inhibit 1-α hydroxylase activities *in vitro* and *in vivo* [31]. Does UA have a similar inhibitory effect on 25 hydroxylase? It is a question that still needs an answer.

Plasma concentrations of 25 OH D and vitamin D-binding globulin (VDBG) were significantly reduced in patients suffering from nephrotic syndrome. VDBG is undetectable in normal urine, but substantial amounts are detected in the urine of nephrotic subjects. Administration of 3H-labeled vitamin D3 to nephrotic subjects resulted in the rapid appearance of labeled 25 OH D3 in the urine bound to VDBG in amounts that largely account for the low plasma labeled 25 OH D3 [32]. Low 25 OH D was also reported in CKD patients having sub-nephrotic range of proteinuria [11] and even in those having microalbuminuria [33]. In the current study, patients with increased urine albumin excretion were in the range of microalbuminuria. Serum 25 OH D in these patients has significant negative association with urine ACR. The molecular weight (MW) of VDBG is 52 kDa [34]. Because of their comparable MW, 25 OH D bound to VDBG is filtered at glomeruli as albumin. Under physiologic conditions, reabsorption of 25 OH D- VDBG complex at proximal tubular cells is mediated by megalin present at the brush border [35]. Whether increased filtration of 25 OH D- VDBG complex or its decreased reabsorption by proximal tubular cells due to down-regulation of megalin in CKD patients with increased UAE still needs further studies. On the other hand, 1,25 (OH)_2_ D -deficient rats develop podocyte injury and renal dysfunction. Expression of nephrin, podocin, and desmin in the podocyte was significantly altered in 1,25 (OH)_2_ D -deficient animals [36]. In addition, glomerular transient receptor potential cation channel 6 (TRPC6) expression is increased in acquired proteinuric renal disease. In 1,25 (OH)_2_ D -deficient mice, TRPC6 expression is increased, accompanied by podocyte foot process effacement and proteinuria [37]. Being the substrate of 1,25 (OH)_2_ D, deficiency of 25 OH D can result in injury of the renal filtration barrier, leading to proteinuria and consequent renal dysfunction.

The highly significant negative impact of serum P looks very interesting. Being the precursor of 1,25 (OH)_2_ D, the correlation between serum 25 (OH)D and serum P was expected to be positive. 1,25 (OH)_2_ D is a strong stimulant of intestinal NPTIIb activity that enhances intestinal phosphate absorption. In CKD patients with impaired renal phosphate excretion, both 25 (OH)D and 1,25 (OH)_2_ D should have positive association with serum phosphorus. The negative association observed in previous studies (12,38) and confirmed in the current study raises many possible mechanisms.

Increased FGF23 level triggered by increased serum P might inhibit 25 hydroxylase or stimulate 24 hydroxylase. FGF23 negatively correlates with 25 OH D in mice, however, similar relation in human CKD patients was not encountered [38,39].

Serum P stimulates PTH synthesis and secretion in CKD [40]. However, PTH stimulates Cyp27b1 responsible for 1-α hydroxylase and inhibits Cyp24a1 responsible for 24 hydroxylase activity [41]. These data make suppression of 25 OH D by PTH unlikely.

The most likely mechanism is a direct effect of P on 25 OH D. Serum P correlates with 24 hydroxylase RNA. This enzyme is responsible for inactivation of 25 OH D into 24,25 OH_2_ D [42]. This means that serum P can decrease 25 OH vit D by stimulating its catabolism.

The current study is observational, rendering a firm conclusion questionable. Further studies that consider patients with higher urine albumin excretion, higher levels of uric acid and followup and interventional trials are still needed to support current results.

## Conclusion and future perspective

These results denote that serum 25 OH D might not be the ideal index of vit D nutritional status among CKD patients. They can also explain the striking association between low serum 25 OH D and vascular calcification [43]. These results should stimulate future studies looking for the impact of control of urine albumin, serum UA and P on serum 25 OH vit D.

## Ethical approval

The local ethical committee of the Internal Medicine Department, School of Medicine, Cairo University, approved this work.

## Human and Animal Rights

All procedures performed in this study involving human participants were in accordance with the ethical standards of the institutional and/or national research committee and with the 1964 Helsinki declaration and its later amendments or comparable ethical standards.

## Informed consent

Informed consent was obtained from all individual participants included in the study.
